# Unconventional non-local relaxation dynamics in a twisted trilayer graphene moiré superlattice

**DOI:** 10.1038/s41467-022-35213-5

**Published:** 2022-12-08

**Authors:** Dorri Halbertal, Simon Turkel, Christopher J. Ciccarino, Jonas B. Profe, Nathan Finney, Valerie Hsieh, Kenji Watanabe, Takashi Taniguchi, James Hone, Cory Dean, Prineha Narang, Abhay N. Pasupathy, Dante M. Kennes, D. N. Basov

**Affiliations:** 1 Department of Physics, Columbia University, New York, NY 10027 USA; 2 Condensed Matter Physics and Materials Science Division, Brookhaven National Laboratory, Upton, NY 11973 USA; 3 Harvard John A. Paulson School of Engineering and Applied Sciences, Harvard University, Cambridge, MA 02138 USA; 4 Institute for Theory of Statistical Physics, RWTH Aachen University, and JARA Fundamentals of Future Information Technology, 52062 Aachen, Germany; 5 Research Center for Functional Materials, National Institute for Materials Science, 1-1 Namiki, Tsukuba, 305-0044 Japan; 6 International Center for Materials Nanoarchitectonics, National Institute for Materials Science, 1-1 Namiki, Tsukuba, 305-0044 Japan; 7 Max Planck Institute for the Structure and Dynamics of Matter, Center for Free Electron Laser Science, Hamburg, Germany

**Keywords:** Two-dimensional materials, Mechanical and structural properties and devices

## Abstract

The electronic and structural properties of atomically thin materials can be controllably tuned by assembling them with an interlayer twist. During this process, constituent layers spontaneously rearrange themselves in search of a lowest energy configuration. Such relaxation phenomena can lead to unexpected and novel material properties. Here, we study twisted double trilayer graphene (TDTG) using nano-optical and tunneling spectroscopy tools. We reveal a surprising optical and electronic contrast, as well as a stacking energy imbalance emerging between the moiré domains. We attribute this contrast to an unconventional form of lattice relaxation in which an entire graphene layer spontaneously shifts position during assembly, resulting in domains of ABABAB and BCBACA stacking. We analyze the energetics of this transition and demonstrate that it is the result of a non-local relaxation process, in which an energy gain in one domain of the moiré lattice is paid for by a relaxation that occurs in the other.

## Abstract (web summary)

In moiré materials, structural relaxation phenomena can lead to unexpected and novel material properties. Here, the authors characterize an unconventional non-local relaxation process in twisted double trilayer graphene, in which an energy gain in one domain of the moiré lattice is paid for by a relaxation that occurs in the other.

## Introduction

The discovery of superconductivity in rotationally misaligned graphene bilayers established moiré engineering as a robust way to create strongly correlated phases in van der Waals heterostructures^1,2^. Since this initial discovery, a wide range of moiré materials have emerged with fascinating electronic properties such as correlated insulators^3–8^, strange metals^9,10^, electronic nematics^11–14^, and Wigner crystals^15^, among other unconventional phases. Moiré materials additionally hold promise as platforms for tunable quantum simulation, enabling future applications in materials discovery and design^16^. With the advent of new and more complex moiré device geometries, with greater than two layers^5–8^ or greater than one twist angle between layers^17–20^, it becomes increasingly important to consider the effects of lattice relaxation, or the spontaneous rearrangement of atoms in search of a lower energy configuration, on the final microscopic crystal structure. In mirror symmetric twisted trilayer graphene, for instance, it was recently observed^21^ that lattice relaxation leads to the emergence of moiré defects that are not observed in the simpler twisted bilayer system. A fuller understanding of relaxation phenomena in moiré heterostructures thus holds the potential to enable exploitation of these effects as a means of engineering novel or otherwise unstable material systems.

**Fig. 1:**
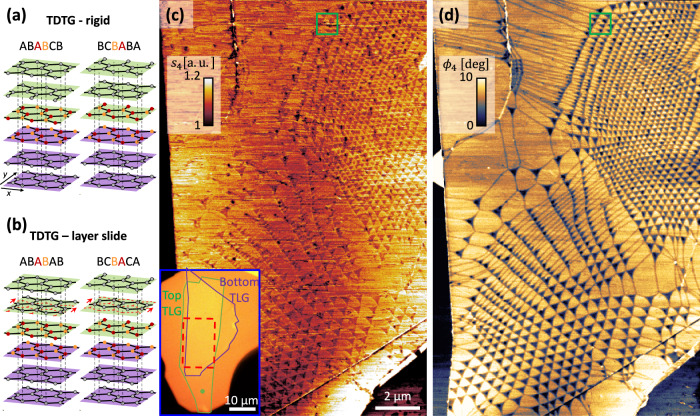
Moiré superlattice of twisted double trilayer graphene (TDTG). **a** The lattice structure of the two lowest energy stacking configurations of TDTG when assuming both top and bottom trilayer graphene (TLG) are Bernal stacked. The interfacial atoms are colored to highlight the AB/BA stacking configurations. **b** The TDTG system after a global slide of the middle layer of one of the TLG sheets. The direction of slide is marked by red arrows indicating the transition from an ABA (dashed red honeycomb lattice) to a BAB configuration. Such a slide, if realized, results in the formation of the unstable BCBACA phase. **c**, **d** Mid-IR near-field amplitude (**c**) and phase (**d**) of the TDTG stack. Bright (convex) and dark (concave) triangular domains are observed across the sample. Near-field signal in **c**, **d** is the 4th harmonic demodulation of the probe tapping frequency (laser illumination at 1000 cm^−1^, see “Methods” for additional experimental details). Inset of **c**: Optical image of the TDTG/hBN stack. The bottom and top TLGs are highlighted by purple and green frames, respectively. The red rectangle marks the scan area of (**c**, **d**).

Twisted double trilayer graphene (TDTG), a moiré material that has not yet been experimentally investigated, is a natural next step in extending the moiré paradigm to more complex structures, in which lattice relaxation can lead to unexpected atomic configurations. TDTG is formed in a manner analogous to twisted bilayer (TBG) and twisted double bilayer graphene (TDBG), namely by stacking two Bernal trilayers from the same source crystal with a small relative twist. In the low twist angle limit, moiré patterns in few-layer graphene (FLG) systems generate large domain structures with distinct crystallographic stackings^22,23^. If rigidly stacked in a manner that preserves the ABA stacking of each trilayer (referred to here as the rigid scenario), TDTG forms domains of mixed rhombohedral and Bernal character with ABABCB and BCBABA stackings (Fig. 1a), where each domain contains a unit of three rhombohedrally stacked layers (3R).

It is conceivable, however, that stresses and strains applied to a sample during the fabrication process can act as an effective annealing, allowing the system to explore other stacking configurations before reaching the lowest energy equilibrium. In the case of TDTG, applying a simple translation to the second layer (Fig. 1b) results in one domain (ABABAB) with pure Bernal stacking and another domain (BCBACA) with a unit of four rhombohedrally stacked layers (4R). This layer slide scenario provides an interesting test case from an energy standpoint. While the pure Bernal phase is energetically favorable, its realization comes at the price of increasing the local stacking energy of the mixed rhombohedral domain by transforming it from a 3R to a 4R configuration. Prior studies of relaxation effects in twisted van der Waals heterostructures^22,24–27^ have focused on processes in which lattice relaxation acts to uniformly reduce the stacking energy at every location in space, generally by minimizing the area of higher energy domains and maximizing the area of their lower energy counterparts, as occurs in minimally twisted TDBG. Consideration of the scenarios presented in Fig. 1a, b for TDTG raises the question of whether relaxation processes can likewise act to offset an increase of the stacking energy in one area of a device with a decrease in an area that is as far as several microns away. Such relaxation at a distance would offer a means of stabilizing elusive atomic configurations with distinct electronic properties by structurally coupling them to low-energy phases through moiré patterning.

## Results

In this work, we utilize mid-infrared scanning near-field optical microscopy (SNOM) and scanning tunneling microscopy (STM) and spectroscopy (STS) to characterize the optical and electronic properties of TDTG samples. The use of SNOM and STM/S on identical samples allows us to characterize the electronic properties of a device over both large (microns) and small (nanometers) areas, giving direct experimental access to the interplay of length scales that is crucial to non-local relaxation dynamics. Figure 1c, d shows phase resolved SNOM images^28–30^ taken over a ~10 μm region of the TDTG device shown in the inset of Fig. 1c. In the rigid scenario for TDTG (Fig. 1a), we expect the local stacking configuration in each domain to be ABABCB and BCBABA, which are related to each other by inversion across the *x*–*y* plane (${C}_{2}^{z}$). In the absence of external ${C}_{2}^{z}$ symmetry breaking mechanisms, such as displacement field or an asymmetric dielectric environment, these two configurations are therefore expected to possess identical electronic and structural properties. Contrary to this expectation, a moiré superlattice with clear optical contrast between adjacent domains is observed both in the amplitude (Fig. 1c) and phase (Fig. 1d) of the near-field signal. Furthermore, there is a clear imbalance in the stacking energy of the two domains, as evidenced by the convexity (concavity) of the bright (dark) triangles in Fig. 1d. This difference in stacking energy is particularly clear in regions of large heterostrain, such as in the top left of Fig. 1d, where a linear pattern is observed, corresponding to double domain walls (DDWs) that emerge due to the collapse of unstable domains^22^.

**Fig. 2:**
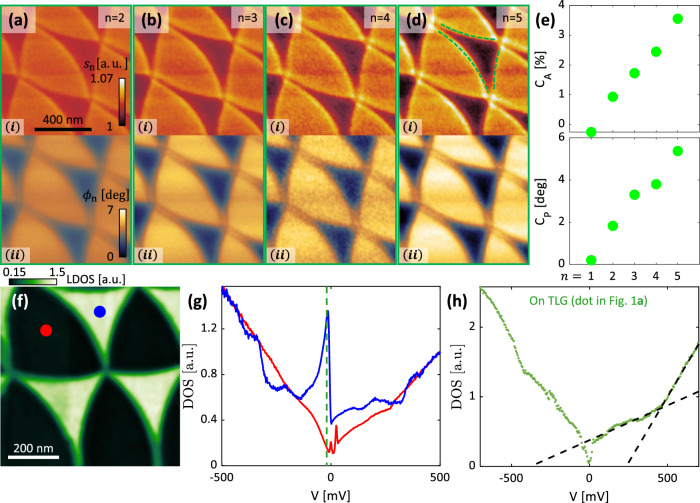
Imaging of the TDTG moiré. **a**–**d** Mid-IR near-field imaging (over green square in Fig. 1a) showing bright (convex) and dark (concave) domains: normalized amplitude (*i*) and phase (*ii*) at different probe demodulation harmonics from 2nd (**a**) to 5th (**d**). The measurement was done at room temperature and a laser frequency of 1000 cm^−1^. **a**–**d** share a color-bar and scale-bar. Dashed arcs in (**d**) with a radius of 850 nm highlight the domain shape. **e** Amplitude (top) and phase (bottom) contrasts between the two domains of (**a**–**d**) as a function of demodulation harmonics, revealing a monotonic trend. **f** Differential conductance measured by STS, acquired at a sample bias of −20 meV. **g** Tunneling spectrum measured in each of the two domains (marked by correspondingly colored dots in (**f**)). The dashed line marks the energy at which (**f**) was acquired. Each curve is normalized to its value at 500 mV. **h** Tunneling spectrum measured on exposed TLG section (green dot in Fig. 1a) indicative of a Bernal stacked TLG source crystal.

Figure 2 examines the TDTG moiré superlattice at the sub-micron length scale. The complex near-field signal is presented in Fig. 2a–d as a function of tapping-probe demodulation harmonic (focusing on the green square in Fig. 1c, d). These finer scans further demonstrate the optical contrast between adjacent domains as well as the curving of the domain walls (DWs). Additional details emerge in these higher-resolution images as well, including clear bright features along the DWs and bright spots at the DW intersections in Fig. 2d. Furthermore, we find that the complex near-field optical contrast between the domains is a monotonically increasing function of demodulation harmonic (Fig. 2e), indicating that the signal is coming from the graphene device rather than the substrate. We examine the microscopic electronic structure in greater detail by performing STM/S measurements over regions of the same TDTG sample. The local density of states (LDOS) measured near the Fermi level is displayed in Fig. 2f, which shows a striking contrast in tunneling conductivity between different domains of the moiré lattice. Characteristic spectra acquired on each of the two domains are plotted in Fig. 2g, demonstrating that the observed LDOS contrast is caused by a large spectral peak near zero energy that is present in only the convex domain. The spectral shape in the concave domain, on the other hand, is largely featureless at low energy and possesses no comparable peak. Moreover, measurements of the tunneling spectrum on the untwisted region (Fig. 2h) indicate that the source crystal is Bernal trilayer, confirming that the moiré contrast is a property of the six-layer system. This suite of measurements unambiguously demonstrates a significant difference in electronic structure between each of the observed moiré superlattice domains. In the Supplementary Information (section 3) we consider and rule out alternative sources of ${C}_{2}^{z}$ symmetry breaking, including the effect of the hBN substrate and the possibility of atomic-scale near-field tomography, i.e., the breaking of ${C}_{2}^{z}$ symmetry by the sharp probe interacting with individual atomic layers. The naive expectation of the rigid scenario (Fig. 1a) is therefore clearly not realized in our TDTG device.

Applying a global translation to one of the six layers in a TDTG heterostructure has the potential to lower the overall stacking energy of a device even as it might raise the energy density in certain confined regions. Once we accept that the energy barrier to such a transition can be overcome (see below), there is in principle no reason to restrict our analysis to the particular layer slide scenario depicted in Fig. 1b. In an effort to match the experimental observations, we have therefore performed DFT calculations of the band structure, density of states, and stacking energy density of all 2^5^ possible TDTG stacking configurations (each of the five-layer interfaces can be stacked as either AB or BA). Eight of these configurations describe, in the minimally twisted regime, moiré pairs that are related by ${C}_{2}^{z}$ symmetry (see Supplementary Information section 4), which we have already ruled out above.

**Fig. 3:**
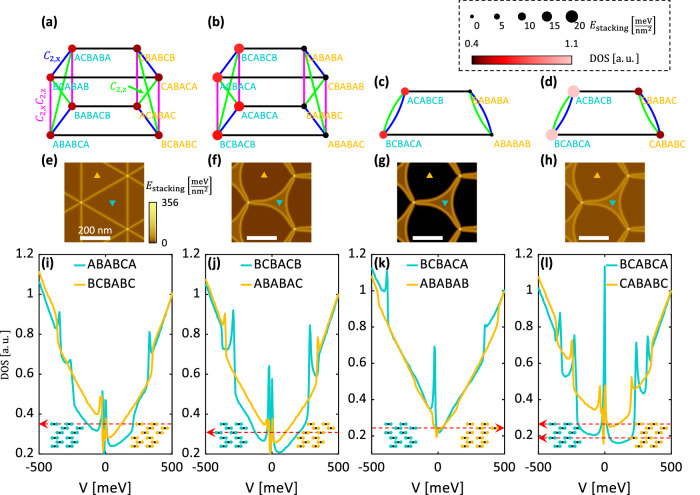
Exploration of candidates for TDTG moiré superlattice structures. **a**–**d** Each panel addresses a group of configurations. Each moiré pair is connected by a horizontal black line. Configurations that are *C*_2_ symmetry pairs are connected by blue (${C}_{2}^{x}$), green (${C}_{2}^{z}$), and magenta (${C}_{2}^{x}{C}_{2}^{z}$) lines, respectively. Each configuration is marked by a circle whose color indicates the low energy spectral weight, and whose size indicates the configuration’s stacking energy density (see legend). **e**–**h** The domain formation is reflected by the stacking energy density for a twist angle of 0.04^∘^ for each moiré pair. Each panel is the result of the atomic relaxation calculation (see “Methods”) using the DFT calculated energy imbalance of the corresponding moiré pair (triangles indicate the phases with matching colors to the configuration text in (**a**–**d**)). **i**–**l** DFT calculated electronic densities of states for different moiré pairs (see “Methods”). The inset shows the lattice structure for each configuration (with consistent colors as in (**a**–**d**)). The red arrow indicates the required global layer sliding in order to realize the particular moiré superlattice from the rigid ABABCB/BCBABA configuration. The configurations where the moiré pairs are also *C*_2_ symmetry pairs were omitted here for brevity, as they could not produce an energy imbalance (these missing configurations are explored in Supplementary Information section 4).

Figure 3 explores the remaining twenty-four TDTG configurations with crystallographically distinct moiré domains. These can be divided into four groups (corresponding to the four columns of Fig. 3) based on their symmetries. Moiré pairs in Fig. 3a–d are connected by black horizontal lines (*C*_2_ symmetry pairs are connected by colored lines as indicated). Each possible moiré domain is characterized by its calculated Fermi level spectral weight and stacking energy density (reflected in Fig. 3a–d by vertex color and size, respectively). Moiré pairs with a large difference in stacking energy result in curved domains, similar to those seen experimentally, as confirmed by atomic relaxation calculations (Fig. 3e–h). In seeking a match with our experimental observations, we therefore require a pair of moiré domains with a large difference in both stacking energy and Fermi level spectral weight to match the domain curvature and electronic contrast revealed by SNOM and STM. While three groups of moiré pairs possess sufficient domain curvature (Fig. 3f–h), only the ABABAB/BCBACA configuration displays calculated spectra that are consistent with our STS results (Fig. 3k). The concave domain of this pair (BCBACA) shows a peak at low energy where the convex domain (ABABAB) remains featureless, as in experiment. In addition, the sharp steps at ~± 350 mV in the BCBACA domain, which are associated with the edges of electronic bands, align quantitatively with similar steps observed experimentally in the concave domain (compare Fig. 2g). This excellent match of the calculated electronic structure with the measured density of states spectrum points conclusively to ABABAB/BCBACA as the stacking configurations spontaneously realized in our TDTG device.

## Discussion

**Fig. 4:**
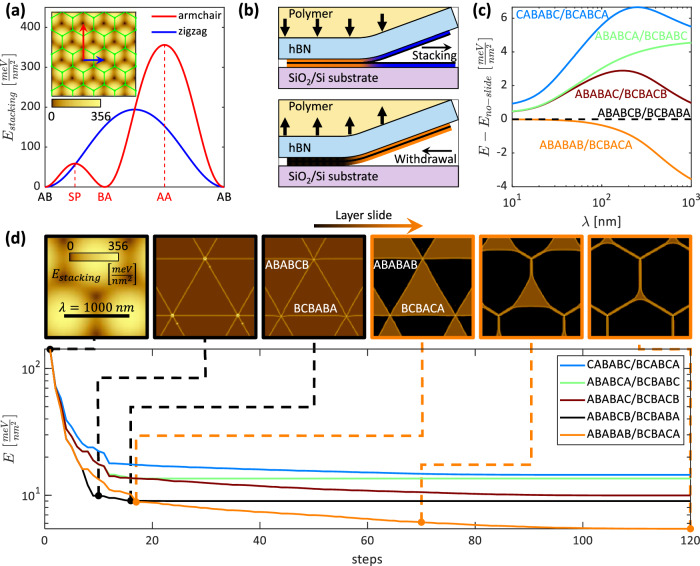
Energy analysis for atomic relaxation driven formation of unstable phases by sliding layers. **a** Stacking energy density at the interface between two graphene sheets for different configurations. The plot presents cuts along the zigzag and armchair directions. Inset: Stacking energy density in the two-dimensional configuration-space reflecting the stacking energy density as a function of translation of one sheet relative to the other (green honeycomb lattice). **b** Scenarios for generation of a new phase during stacking. Top: New phase (orange) generation during contact of the two TLGs (blue). Bottom: New phase generation (orange) as the TDTG stack is picked up from the surface. **c** Comparing total energy density as a function of moiré periodicity for different relaxed moiré superlattices (see “Methods” for details on atomic relaxation models). All curves are referenced to the rigid scenario, ABABCB/BCBABA. ABABAB/BCBACA becomes increasingly energetically favorable as the moiré period increases. **d** Simulating the ABABCB/BCBABA to ABABAB/BCBACA phase stability inversion through the atomic relaxation process. Each curve shows the total energy density for a particular phase at different optimization steps of the gradient-descent process. Above: instantaneous stacking energy densities of the lowest energy moiré system at representative steps. A sharp transition between favorable ABABCB/BCBABA (black) to favorable ABABAB/BCBACA (orange) is observed as the relaxation progresses.

Constructing an ABABAB/BCBACA stacking configuration from a minimally twisted Bernal trilayer source crystal requires a global sliding of the middle layer of one of the two twisted trilayers (illustrated in Fig. 3k, inset). The energetics of such a global translation are seemingly counter-intuitive because, first, it requires a large energy input to the system to realize a universal layer shift, and second, that shift results in a local increase in the stacking energy density of one of the two domains. Sliding of a graphene layer can be viewed as continuously traversing the stacking energy landscape shown in Fig. 4a. To transform adjacent layers from AB to BA stacking, as required to realize the experimentally observed configuration, the system must cross a formidable energy barrier of $\sim 6\times 1{0}^{4}\,\frac{{{{{{{{\rm{eV}}}}}}}}}{\upmu {{{{{{{{\rm{m}}}}}}}}}^{2}}$ set by the saddle-point (SP) of the stacking energy function (indicated by SP in Fig. 4a). This cannot conceivably be overcome by thermal excitation alone. The only step in our experiment during which the sample is subjected to forces of sufficient magnitude to induce a sliding transformation is the stacking process, which involves pressing together each constituent trilayer of the TDTG device before peeling them away from the exfoliation substrate (Fig. 4b). When stacking induced sliding transitions like this have been observed in the past^31^, they have as a rule been from a metastable (rhombohedral) to a stable (Bernal) phase. In our case, however, the transition from ABABCB/BCBABA (3R/3R) to ABABAB/BCBACA (0R/4R) acts to decrease the thermodynamic stability of nearly half of the device area.

Lattice relaxation in TDTG therefore takes an unconventional form in which an energy gain in one half of the crystal is paid for by a relaxation process that occurs in the other. The energy justification for this phenomenon is studied in Fig. 4c, where each curve represents the total energy density (stacking and elastic energy) of a given moiré pair, after atomic relaxation, as a function of moiré wavelength *λ*. All curves are referenced to the energy of the rigid configuration (ABABCB/BCBABA), marked by *E*_*no*−*slide*_. For small *λ* (and therefore weak relaxation) the ABABCB/BCBABA and ABABAB/BCBACA configurations are energetically equivalent, indicating that a layer slide transition without additional relaxation cannot reduce the global energy. As the moiré period increases, the relaxation strengthens, and the energetic imbalance between ABABAB and BCBACA domains (absent in the ABABCB/BCBACA configuration) drives the expansion of the Bernal domain, thus reducing the global energy of the ABABAB/BCBACA configuration relative to its rigid counterpart. If provided sufficient shear forces to overcome the SP energy barrier, the atomic relaxation process can therefore create and stabilize the formation of the otherwise unstable BCBACA phase simply by reducing its relative volume fraction.

We visualize the dynamics of this non-local relaxation in Fig. 4d, where we plot the instantaneous solution to the energy minimization problem for each stacking considered in Fig. 4c as a function of iteration number within the steepest-descent optimization algorithm, which can be interpreted as an effective time coordinate, *t*. At *t* = 0, before any relaxation has taken place, all configurations have similar energies. As the systems flow down their respective energy landscapes, large domains of uniform stacking are formed, separated by straight DWs. In this intermediate regime, the rigid (ABABCB/BCBABA) and layer slide (ABABAB/BCBACA) scenarios have equivalent energies. Only when the relaxation process has reached a point where the DWs begin to curve, with the lower energy Bernal phase pushing into the metastable BCBACA configuration, does a layer slide transition become energetically favorable (see crossing of the black and orange lines in Fig. 4d). Minimally twisted TDTG thus spontaneously seeks a solution in which a transition to a locally metastable phase (BCBACA) is enabled by a shared phase boundary with a proximate stable structure (ABABAB).

The development of new and increasingly complex moiré heterostructures demands a revisiting of some of the basic assumptions of van der Waals engineering. It is sometimes convenient to think of layered materials as immutable building blocks that can be exfoliated and stacked at will. In reality, however, van der Waals materials inhabit a complicated energy landscape that must be carefully navigated when designing new device architectures. Our measurements of minimally twisted TDTG reveal a surprising crystallographic transformation that occurs during the stacking process. The mechanism underlying this transition involves a non-local energy balancing that enables the formation of rhombohedral domains by their coupling to a simultaneously formed relaxed Bernal structure. This has immediate implications both for research and practical applications, as it reveals a heretofore unexplored factor in determining the final structure of fabricated devices. As device geometries become increasingly complex, non-local relaxation phenomena are likely to play an important role in device design. Detailed knowledge of this and similar relaxation processes holds the potential to utilize the power of lattice relaxation for engineering novel and otherwise unstable material systems.

## Methods

### Samples preparation

#### Exfoliation

Graphene and hBN flakes were mechanically exfoliated from the bulk single crystals onto SiO_2_/Si (285 nm oxide thickness) chips using the tape-assisted exfoliation technique (the tape used was Scotch Magic Tape). The Si chips were treated with O_2_ plasma (using a benchtop radio frequency oxygen plasma cleaner of Plasma Etch Inc., PE-50 XL, 100 W at a chamber pressure of 215 mTorr) for 20 s for graphene and no O_2_ plasma treatment for hBN. The chips were then matched with respective exfoliation tape. In the graphene case, the chip+tape assembly were heated at 100^ ∘^C for 60 s and cooled to room temperature prior to removing the tape. Such thermal treatment was not done for hBN.

#### Stack preparation

The heterostructure was assembled using standard dry-transfer techniques^32^ with a polypropylene carbonate (PPC) film mounted on a transparent-tape-covered polydimethylsiloxane (PDMS) stamp. The transparent tape layer was added to the stamp to mold the PDMS into a hemispherical shape which provides precise control of the PPC contact area during assembly^33^. The heterostructure was made by first picking up the hBN (20-nm thick). Prior to pick-up, mechanically exfoliated TLG flakes on Si/SiO_2_ were separately patterned with anodic-oxidation lithography^34^ to facilitate the “cut-and-stack” technique^35^. Next, the PPC film with the heterostructure on top is mechanically removed from the transparent-tape-covered PDMS stamp and placed onto a Si/SiO_2_ substrate such that the final pick-up layer is the top layer. Then the underlying PPC was removed by vacuum annealing at 350 ^∘^C.

### Near-field imaging techniques

The mid-IR near-field scans in this work were acquired with a phase-resolved scattering type scanning optical microscope imaging (s-SNOM) with a commercial system (Neaspec), using a mid-IR quantum cascade laser (Hedgehog by Daylight Solutions) tuned between 8.7 and 10.2 μm. The laser light was focused to a diffraction limited spot at the apex of a metallic tip, while raster scanning the sample at tapping mode. We collect the scattered light (power of 3–5 mW) by a cryogenic HgCdTe detector (Kolmar Technologies). The near-field amplitude and phase were extracted as harmonic components of the tapping frequency using an interferometric detection method, the pseudo-heterodyne scheme, by interfering the scattered light with a modulated reference arm at the detector^28^. The near-field scans of Figs. 1 and 2 were taken at 983 and 1000 cm^−1^, respectively.

### Scanning tunneling microscopy and spectroscopy

STM/S measurements were conducted in a home-built STM under ultra-high vacuum at 7 K. The tungsten tunneling tip was electrochemically etched and calibrated against the Au(111) surface state prior to each sample approach. Spectroscopy was measured using a lock-in amplifier to record the differential conductance with a bias modulation between 1 and 7 mV at 927 Hz, a set point voltage of 250 mV, and a set point current of 120 pA.

### Electronic structure theory calculations of generalized stacking fault energy function (GSFE) and DOS

In order to capture the generalized stacking fault energy function (GSFE) and electron density of states (DOS), we rely on density functional theory calculations. The different six-layer graphene stacking configurations were captured within a hexagonal unit cell with an in-plane lattice constant of *a* = *b* = 2.459 Å. We describe the system using a 24 × 24 × 1 k-point mesh within the plane-wave code JDFTx^36^. Fermi smearing of width 0.01 Hartree is applied to the electronic occupations. We use ultrasoft pseudopotentials^37^ and the PBEsol exchange-correlation functional^38^. In order to remove any artificial interactions between periodic images in the out-of-plane direction, we use a Coulomb truncation technique^39^. The plane-wave cutoff used is 40 Hartrees. We use a stringent charge density cutoff of 1000 Hartrees in order to densely sample the *z* direction of the unit cell, which is important for accurately describing the energetics of the different stacking configurations and therefore for comparison among them. Van der Waals interactions between the graphene layers are modeled using the DFT-D3 scheme^40^.

Using these calculations as starting points, we can then capture the electronic density of states. We describe the electronic states of our systems using a real-space Wannier representation based on maximally-localized Wannier functions^41^. This allows us to sample the electronic energies at arbitrary wave vectors. In our DOS calculations, we sample 5.76 × 10^7^ wave vectors to accurately converge the DOS. We use a Lorentzian with a broadening of width 4.3 meV to smooth the results.

### Atomic relaxation calculations

Modeling of the atomic relaxation of TDTG structures was performed within a continuity model^22,42^. In this model, the total energy of the system is taken as the sum of elastic energy and a stacking energy term. The total energy was minimized in search for the inter-layer real space displacement field corresponding to the relaxed structure. The stacking configuration at the interface was imposed to be AA at the four corners of the moiré unit-cell. The mechanical relaxation parameters (bulk and shear moduli) for TLG as well as the generalized stacking fault energy function (GSFE) for the TLG/TLG interface were calculated using DFT (see DFT section in “Methods” for details). The resulting mechanical coefficients for TLG (in meV per unit-cell) are: bulk modulus—*K* = 210,971, shear modulus—*G* = 151,580.

The GSFE coefficients were extracted from a 7 × 7 sampling of the configuration between two *ABA*-TLG, with the vertical positions of the atoms relaxed at each configuration. The Fourier components of the resulting energies were then extracted to create a convenient functional form for the GSFE used to describe the stacking energy term in the atomic relaxation calculations. For simplicity, the GSFE for configurations other than the nominal case (ABABCB/BCBABA) used the extracted GSFE for the nominal case while imposing the stacking energies at the lowest energy configurations as calculated by DFT (values are detailed in Supplementary Table 1). The GSFE for a given configuration was taken as the closest curve (*L*_2_ norm) with the same functional structure, that satisfies the imposed lowest energy configuration. This approach circumvented the need to calculate the full stacking landscape for all systems. The validity of this approach was assessed comparing the resulting GSFE with the full GSFE calculation for BCABCA/CABABC yielding similar results. The resulting GSFE coefficients are detailed in Supplementary Table 2.

### Supplementary information

Supplementary Information

## Data Availability

The data used in this study are available in the Harvard Dataverse database [10.7910/DVN/VDKZMA].
