# Supplementary material for: Unconventional non-local relaxation dynamics in a twisted trilayer graphene moiré superlattice
Source: Nat Commun. 2022 Dec 8;13:7587. doi: 10.1038/s41467-022-35213-5 (PMC9731949; doi:10.1038/s41467-022-35213-5)
Supplement: Supplementary file 1 — Supplementary Information [file 41467_2022_35213_MOESM1_ESM.pdf]

Supplementary Information for

Unconventional Non-local Relaxation Dynamics in a Twisted Graphene Moiré Superlattice

Dorri Halbertal[1,*,†], Simon Turkel[1,2,†], Christopher J. Ciccarino[3], Jonas Hauck[4], Nathan Finney[1], Valerie Hsieh[1], Kenji Watanabe[5], Takashi Taniguchi[6], James Hone[1], Cory Dean[1], Prineha Narang[3], Abhay N. Pasupathy[1,2], Dante M. Kennes[4,7], and D. N. Basov[1]

[1] Department of Physics, Columbia University, New York, NY 10027, USA
[2] Condensed Matter Physics and Materials Science Division, Brookhaven National Laboratory, Upton, NY 11973, USA
[3] Harvard John A. Paulson School of Engineering and Applied Sciences, Harvard University, Cambridge, MA 02138, USA
[4] Institute for Theory of Statistical Physics, RWTH Aachen University, and JARA Fundamentals of Future Information Technology, 52062 Aachen, Germany
[5] Research Center for Functional Materials, National Institute for Materials Science, 1-1 Namiki Tsukuba 305-0044, Japan
[6] International Center for Materials Nanoarchitectonics, National Institute for Materials Science, 1-1 Namiki Tsukuba 305-0044, Japan
[7] Max Planck Institute for the Structure and Dynamics of Matter, Center for Free Electron Laser Science, Hamburg, Germany

[*]Correspondence to: dorrihal@gmail.com
[†]These authors contributed equally

1 Calculation of the optical conductivity

To calculate the optical conductivity of graphene multilayers, we begin with the simplest possible tight-binding model Hamiltonian, which has proven to describe the optical properties of few-layer Graphene well[47,48]. This model consists of two hopping terms, the nearest-neighbor in-plane hopping $\gamma_0 = 3.16$ eV and the nearest-neighbor out-of-plane hopping $\gamma_1 = 0.39$ eV. All other hopping terms are neglected as they mainly alter the low energy region, which we are not accessing in the experiment. To consider the effect of electric fields on the measurement, we performed simulations in two different extreme settings. For the first case, we assumed that the outermost layers fully screen the applied field. This is incorporated into the Hamiltonian by a shift of $\pm\frac{e\phi}{2}$ of the chemical potential of the two outermost layers. For the second case, we assumed that each layer screens away the same amount of the field. This results in a decrease by $\frac{e\phi}{N}$ (where N is the total number of layers: $N = 6$ for TDTG) from layer to layer, such that the uppermost lies at $\frac{e\phi}{2}$- and the lowermost at $-\frac{e\phi}{2}$-potential.

Once the tight-binding Hamiltonian is established we employ the standard Kubo formalism to calculate the optical conductivity in the linear response regime[49]. In the following we will sketch the steps necessary to derive this formula. We use atomic units for convenience. The starting point for the derivation is a general non-interacting tight-binding Hamiltonian

$$\hat{H} = \sum_{\mathrm{i,j},\sigma} t_{\mathrm{i,j}} c^{\dagger}_{\mathrm{i},\sigma} c_{\mathrm{j},\sigma} = \sum_{\mathrm{m,n},\sigma,k} \sum_{R,i,j} t_{\mathrm{i,j}} \delta_{r_{\mathrm{m}}+R, r_{\mathrm{n}}+d_{\mathrm{i,j}}} e^{-ik\cdot R} c^{\dagger}_{\mathrm{m},\sigma}(k) c_{\mathrm{n},\sigma}(k), \tag{1}$$

with $t_{\mathrm{i,j}}$ the tight binding hopping elements each associated with a vector $d_{i,j}$ connecting two lattice sites. The operators $c^{(\dagger)}_{\mathrm{i},\sigma}$ annihilate (create) an electron on site i with spin σ and $c^{(\dagger)}_{\mathrm{m},\sigma}(k)$ annihilate (create) an electron on unit-cell site m with spin σ and momentum k. i, j denote positions in the full lattice and m, n denote positions within the unit-cell. The basis vectors spanning the real lattice are denoted as R. If we illuminate our sample with a weak laser we can incorporate it by performing a Peierls substitution

$$t_{\mathrm{i,j}} \to t_{\mathrm{i,j}} e^{iA(t)\cdot\delta_{\mathrm{i,j}}}, \tag{2}$$

where the time dependent vector potential $A(t)$ has been introduced. The physical charge current generated by this perturbation can be extracted from a second order Taylor expansion in one of the three spatial directions α of the exponential and is given by

$$\hat{\mathscr{J}}_{\alpha} = -\frac{\partial \hat{H}}{\partial A_{\alpha}(t)} = +\hat{j}_{\alpha}|_{A(t)=0} + A_{\alpha}(t)\hat{j}^{D}_{\alpha}|_{A(t)=0}. \tag{3}$$

Here, the diamagnetic current is defined as

$$\hat{j}^{D}_{\alpha} = \sum_{\mathrm{m,n},\sigma,k} \sum_{R,i,j} (d_{\mathrm{i,j}})^2_{\alpha}\, t_{\mathrm{i,j}} \delta_{r_{\mathrm{m}}+R, r_{\mathrm{n}}+\delta_{\mathrm{i,j}}} e^{-ik\cdot R} c^{\dagger}_{\mathrm{m},\sigma}(k) c_{\mathrm{n},\sigma}(k) \tag{4}$$

and the normal current as

$$\hat{j}_{\alpha} = -i \sum_{\mathrm{m,n},\sigma,k} \sum_{R,i,j} (d_{\mathrm{i,j}})_{\alpha}\, t_{\mathrm{i,j}} \delta_{r_{m}+R, r_{\mathrm{n}}+\delta_{\mathrm{i,j}}} e^{-ik\cdot R} c^{\dagger}_{\mathrm{m},\sigma}(k) c_{\mathrm{n},\sigma}(k). \tag{5}$$

From linear response theory we know that the optical conductivity is given by

$$\sigma_{\alpha,\beta}(z) = \frac{i}{z} \langle \hat{j}^{D}_{\alpha} \rangle \delta_{\alpha,\beta} + \frac{1}{zV} \int_0^{\infty} dt\, e^{izt} \langle [\hat{j}_{\alpha}(t)_H, \hat{j}_{\beta}] \rangle\,. \tag{6}$$

The first term can be evaluated straight forward. For the second term, we assume that we know a diagonal basis of the Hamiltonian, whose eigenstates we label with q, p. This allows us to write

$$\int_0^\infty dt\, e^{izt} \langle [\hat{j}_\alpha(t)_H, \hat{j}_\beta] \rangle = \sum_{\mathrm{p,q}} j_\alpha^{pq} j_\beta^{qp} \int_0^\infty dt\, e^{izt} e^{i(\varepsilon_p - \varepsilon_q)t} \langle c_p^\dagger c_q c_q^\dagger c_p - c_p^\dagger c_q c_q^\dagger c_p \rangle \tag{7}$$

$$= -i \sum_{\mathrm{p,q}} j_\alpha^{pq} j_\beta^{qp} \frac{n_f(\varepsilon_p) - n_f(\varepsilon_q)}{z + \varepsilon_p - \varepsilon_q}. \tag{8}$$

Collecting all contributions and performing an analytic continuation to $z = \omega + i\eta$ gives

$$\sigma_{\alpha,\beta}(\omega + i\eta) = \frac{i}{\omega + i\eta} \sum_q j_{q,q}^D n_f(\varepsilon_q) \delta_{\alpha,\beta} - i \sum_{\mathrm{p,q}} \frac{1}{(\omega + i\eta)V} j_\alpha^{pq} j_\beta^{qp} \frac{n_f(\varepsilon_p) - n_f(\varepsilon_q)}{(\omega + i\eta) + \varepsilon_p - \varepsilon_q} \tag{9}$$

$$= \frac{i}{\omega + i\eta} \sum_q j_{q,q}^D n_f(\varepsilon_q) \delta_{\alpha,\beta} - \frac{i}{V} \sum_{\mathrm{p,q}} \frac{n_f(\varepsilon_p) - n_f(\varepsilon_q)}{\varepsilon_p - \varepsilon_q} j_\alpha^{pq} j_\beta^{qp} \left(\frac{1}{(\omega + i\eta) + \varepsilon_p - \varepsilon_q} - \frac{1}{(\omega + i\eta)} \right). \tag{10}$$

At low frequencies, the real part of this equation is governed by Lorentzian contributions, which can be associated to the Drude weight of the system. Relabeling the eigenstates p, q by band and momentum indices we arrive at

$$\begin{aligned} \sigma_{\alpha,\beta}(\omega) = & \frac{i}{\omega + i\eta} \sum_{b_1,k} j_{b_1,b_1}^D(k) n_f(\varepsilon_{b_1}^k) \delta_{\alpha,\beta} \\ & - \frac{i}{V} \sum_{b_1,b_2,k} \frac{n_f(\varepsilon_{b_1}^k) - n_f(\varepsilon_{b_2}^k)}{\varepsilon_{b_1}^k - \varepsilon_{b_1}^k} j_\alpha^{b_1,b_2}(k) j_\beta^{b_2,b_1}(k) \left(\frac{1}{(\omega + i\eta) + \varepsilon_{b_1}^k - \varepsilon_{b_1}^k} - \frac{1}{(\omega + i\eta)} \right). \end{aligned} \tag{11}$$

As we have a layered structure we need to identify the optical sheet conductivity of each layer. For this purpose, we define the layered current operator according to

$$j_\alpha^{b_1,b_2}(k)_{layer} = \frac{1}{2} \left\langle \varepsilon_{b_1}^k \middle| n^k \right\rangle \left(j_{n,m}(k) \delta_{z_{layer},z_m} + j_{n,m}(k) \delta_{z_{layer},z_n} \right) \left\langle m^k \middle| \varepsilon_{b_2}^k \right\rangle \tag{12}$$

Where we introduced two momentum-orbital-space unities $|m^k\rangle \langle m^k|$. In this basis, m labels the sites within the unit cell, therefore we can identify to which layer each site belongs and restrict the current operator to this specific layer. The layered conductivity τ_n is now obtained by introducing the definition (12) in each term once:

$$\begin{aligned} \tau_n^{\alpha,\beta}(\omega) = & \frac{i}{\omega + i\eta} \sum_{b_1,k} j_{b_1,b_1}^D(k)_n\, n_f(\varepsilon_{b_1}^k) \delta_{\alpha,\beta} \\ & - \frac{i}{V} \sum_{b_1,b_2,k} \frac{n_f(\varepsilon_{b_1}^k) - n_f(\varepsilon_{b_2}^k)}{\varepsilon_{b_1}^k - \varepsilon_{b_1}^k} j_\alpha^{b_1,b_2}(k)_n\, j_\beta^{b_2,b_1}(k) \left(\frac{1}{(\omega + i\eta) + \varepsilon_{b_1}^k - \varepsilon_{b_1}^k} - \frac{1}{(\omega + i\eta)} \right). \end{aligned} \tag{13}$$

These layer conductivities (defined in eq. 13) sum to the full conductivity. We chose $T = 0.025\,\mathrm{eV}$ and a phenomenological broadening $\eta = 30\,\mathrm{meV}$. In practice we represent the conductivity in units of $\sigma_0 = \frac{\pi e^2}{2h}$, the optical conductivity of a single layer graphene.

2 Evidence the source crystal is Bernal trilayer graphene

Prior to device fabrication, the thickness of the source graphene crystal was determined using its optical contrast against the SiO_2 exfoliation substrate in the standard way (Supplementary Fig. 1a, inset). We checked that the source crystal was Bernal rather than rhombohedral by performing Raman spectroscopy on several areas. The Raman 2D-mode of few layer graphene exhibits distinctive features depending on the stacking configuration, with rhombohedral graphene showing a pronounced asymmetric line shape[47,50]. In our Raman measurements we observe no features indicative of rhombohedral graphene, confirming that our source crystal was stacked in the Bernal configuration (Supplementary Fig. 1a). We further confirmed the thickness of the source crystal as trilayer by measuring STM topography across the step leading to the twisted region of the device (Supplementary Fig. 1b). Spectroscopy measured below the step provides additional evidence that the source crystal was Bernal stacked, as described in the main text (Fig. 2h).

3 Alternative mechanisms for the moiré contrast and feasibility of atomic-scale near-field tomography

The observation of strong contrasts in the optical conductivity and the local density of states, and the difference in structural stability between the moiré domains in TDTG implies either that the stacking configurations in our device follow the rigid scenario (Fig. 1a) and that an additional C_2^z symmetry breaking mechanism is present, or that the layer slide scenario (Fig. 1b) is realized through a non-local relaxation effect. We have considered several possible C_2^z symmetry breaking mechanisms in an attempt to explain the observed moiré contrast. One source of C_2^z symmetry breaking could have been an out-of-plane displacement field caused by trapped charge in the substrate. We can rule this out because such substrate inhomogeneities would likewise dope the graphene away from charge neutrality, which would in turn be clearly visible in STS measurements as an energy shift of the charge neutrality point. In our measured spectrum on exposed trilayer (Fig. 2h), however, the minimum in the density of states is pinned to zero energy, indicating negligible intrinsic doping and hence negligible displacement field. Another potential source of C_2^z symmetry breaking is the asymmetric dielectric environment imposed by the substrate itself, since the presence of hBN on one side of the device and vacuum on the opposite side does in principle break C_2^z symmetry. To investigate whether this asymmetry can lead to significant differences in the electronic properties or stacking energies of the two domains we have performed first principles Density Functional Theory (DFT) calculations (see Methods for details) and found that the hBN substrate introduces an energy imbalance of at most 70 μeV/nm^2, which would produce a DW radius of curvature of over 40 μm. The true radius of curvature as measured in SNOM and STM is $\sim$ 850 nm (see overlaid arcs in Fig. 2d), an order of magnitude less than that predicted due to the substrate effect, ruling out the latter as an explanation of our data.

The only remaining plausible C_2^z symmetry breaking mechanism in our experiments is in the geometry of the scanned probes themselves. In both SNOM and STM, the signal is collected from a metallic probe that is suspended above the sample. As a result, measurements of multilayer samples tend to be most sensitive to the properties of those layers that are physically closest to the probe. In STM, this can lead to exponential sensitivity to the density of states of the topmost layer due to the rapid decay of the surface wave function across the vacuum tunneling barrier[11,23]. The SNOM signal, on the other hand, is generated by a classical electric field that follows a power law decay, which makes it possible to perform tomographic imaging of bulk materials by controlling the height of the probe[51–53]. This asymmetric probe sensitivity has been exploited to attain imaging resolution along the vertical (z) direction, using a technique called near-field tomography (NFT). NFT utilizes the fact that the z-profile of the complex optical conductivity is

imprinted in the near-field probe approach curve[51,54]. NFT was recently used, for example, to decouple surface and bulk contributions to the near-field signal in a topological insulator[53].

Until now it has been assumed that the vertical resolution of NFT is limited by the curvature of the AFM tip, which is typically several tens of nanometers. This limitation arises from the radius of curvature setting the decay length of the electromagnetic (EM) field away from the tip. If, however, a device architecture can be engineered so as to reduce or eliminate far field contrast, then the resolution of NFT can be significantly enhanced. In fact, we find that a heterostructure consisting of ABABCB and BCBABA domains consitutes just such a system, in which the vertical resolution of NFT can be boosted to sub-nanometer length scales. We define a conductivity τ_i for layer $i = 1,...,6$ (Supplementary Fig. 2a), consistent with the Hamiltonian of the system (Supplementary Information section 1). From this definition the ABABCB and BCBABA phases are modelled by stacking τ_i with the appropriate layer order, separated by the layer spacing of graphite (Inset of Supplementary Fig. 2a). The observed near-field contrast is uniquely generated by an EM z-gradient and will not have any contribution from a uniform EM field, strictly due to the C_2^z symmetry relation between ABABCB and BCBABA stackings. Plugging the optical conductivities of Supplementary Fig. 2a into the lightning rod model solver[55] (LRM) we get small differences between the ABABCB and BCBABA approach curves (Supplementary Fig. 2b). After demodulation, these approach curves result in a near-field contrast for different probe harmonics (Supplementary Fig. 2c) well within our measurement capabilities (Fig. 2e). We emphasize that the ability to resolve the atomic scale tomographic differences between ABABCB and BCBABA stacking configurations is a direct consequence of the C_2^z symmetry relation between these domains, which eliminates all far field optical contrast. While we can rule out NFT as a description of our experimental results, as described in the main text, the foregoing demonstrates that NFT at the atomic scale is in principle achievable in device geometries with mirror symmetric designs.

4 Possible TDTG configurations where the moiré superlattice domains are C2 symmetry pairs

Here, we consider the eight stacking configurations for TDTG that were omitted in the main text. These eight configurations, displayed in Supplementary Fig. 3, constitute four moiré pairs that are related by C_2^z symmetry. Unsurprisingly, these moiré pairs exhibit no contrast in stacking energy or in electronic structure, and therefore cannot be accurate descriptions of the stacking configurations realized in our experimental device.

5 The optical contrast of the moiré superlattice in TDBG

Several prior experimental works have reported near-field optical contrast between domains of Bernal (ABAB) and rhombohedral (ABCA) few layer graphene[22,23], similar to the contrast between ABABAB and BCBACA domains observed in the present work (Fig. 1). This observed contrast is typically attributed to differences in the optical conductivity between Bernal and rhombohedral graphenes. Such an attribution is plausible, given the significant difference in electronic structure between the two stacking configurations, however it has not yet been justified from a theoretical perspective. Here, we demonstrate that the observed near-field contrast in domains of marginally twisted double bilayer graphene is quantitatively consistent with the calculated optical conductivities of the ABAB and ABCA stacking configurations.

Supplementary Fig. 4a,b illustrates the contrast in amplitude and phase observed in SNOM measurements of TDBG. In these images, the darker (brighter) triangles correspond to ABCA (ABAB) stacking,

as evidenced by the concavity (convexity) of their edges. In order to explain this contrast, we use the tight binding derived band structures of the ABAB and ABCA stacking configurations (see Supplementary Information section 1) to compute the frequency dependent complex optical conductivity of each domain, shown in Supplementary Fig. 4c. We then simulate the near-field signal with the lightning rod model (LRM)[55], using a tip radius of 30 nm and tapping amplitude of 40 nm for each stacking configuration. The LRM calculation assumes that each stacking configuration is placed on a 40 nm thick hBN substrate on top of 285 nm thick SiO_2 on Si. This results in a frequency dependent amplitude and phase for each domain. To facilitate comparison with experiment, we examine the amplitude and phase contrast between ABCA and ABAB domains within the LRM calculation, shown in Supplementary Fig 4d for the lowest five tapping harmonics. The frequency at which the experimental images in Supplementary Fig. 4a,b were acquired is marked with a vertical dashed line. The calculated contrast in both the amplitude and phase channels shows good quantitative agreement with the measured contrast, demonstrating that the contrast observed in the experimental images can be generated by the different band structures and thus optical conductivities of the two stacking configurations.

Table 1. Stacking energy for the two lowest energy configurations for different moiré systems (in units of $\frac{\text{meV}}{\text{nm}^2}$).

**ABABCB**	5.059	**BCBABA**	5.059
**ABABCA**	9.755	**BCBABC**	9.883
**BCBACB**	14.746	**ABABAC**	4.768
**BCBACA**	10.042	**ABABAB**	0
**BCABCA**	19.420	**CABABC**	9.553

Table 2. GSFE parameters ($c_{0,\ldots,5}$ - following nomenclature of Ref. 46 and units of $\frac{\text{meV}}{\text{nm}^2}$) for different moiré configurations considered in this work.

	c_0	c_1	c_2	c_3	c_4	c_5
**ABABCB/BCBABA**	141.38	81.94	-7.55	-2.79	0	0
**ABABCA/BCBABC**	142.14	80.61	-5.62	-3.65	-0.0152	-0.0095
**BCBACB/ABABAC**	142.13	80.62	-5.64	-3.64	1.179	0.742
**BCBACA/ABABAB**	141.76	81.26	-6.57	-3.23	1.186	0.747
**BCABCA/CABABC**	142.50	79.98	-4.71	-4.05	1.166	0.734

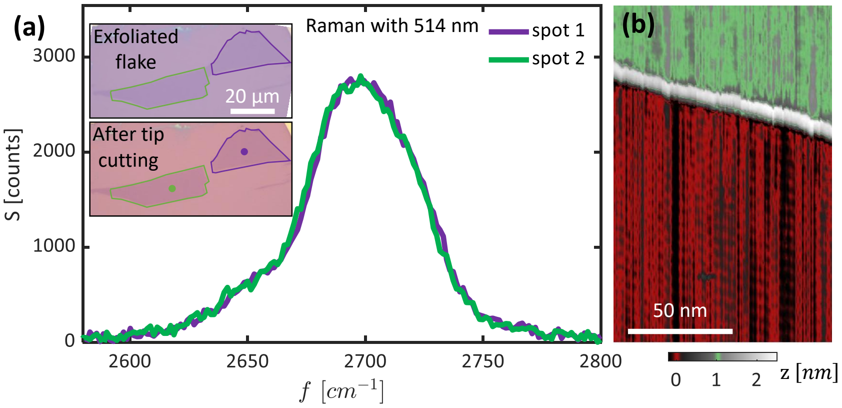

Supplementary Fig. 1. Evidence the source crystal is Bernal trilayer graphene. **(a)** Raman spectra of the 2D-mode acquired on two different spots of the source crystal with 514 nm laser wavelength. Inset: optical micrographs of the source crystal before (top) and after (bottom) AFM cutting. Blue and green spots indicate where Raman spectra were obtained. **(b)** STM topograph of the fabricated TDTG stack showing a trilayer step at the edge of the twisted region.

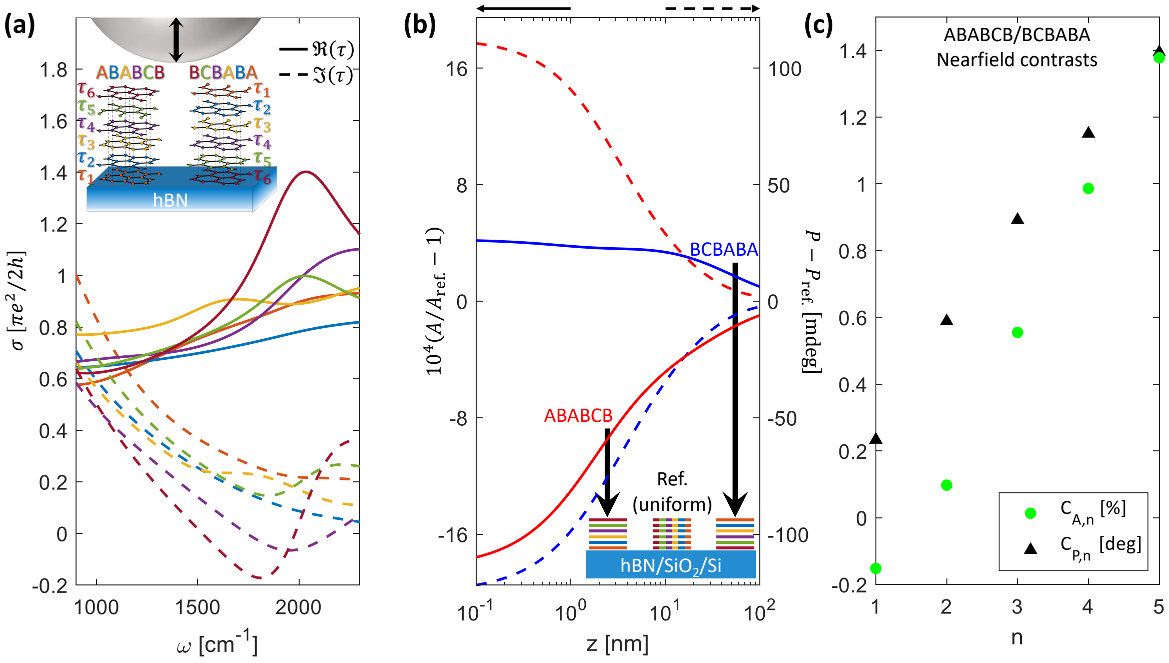

Supplementary Fig. 2. Atomic scale near-field tomography in ABABCB/BCBABA TDTG. (a) Real (solid) and imaginary (dashed) parts of the layer resolved complex optical conductivity (τ_i) for each of the six layers of ABABCB stacked graphene. Layer conductivity assumes $E_F = 60$ meV and a 5 meV broadening (see Supplementary Information section 1 for additional details). Inset: schematic of near-field tomography showing that the enhanced localized field under the tapping probe (not to scale) breaks C_2^z symmetry and generates a contrast in the near-field response. **(b)** Comparing amplitude (left, solid) and phase (right, dashed) of approach curves. The ABABCB (red) and the BCBABA (blue) approach curves are presented with respect to a reference stack with similar total conductivity but distributed uniformly across the layers (see Supplementary Information section 1 for calculation details). Inset: schematics of the three discussed configurations; colors match those in **(a)**. **(c)** The ABABCB/BCBABA contrast as a function of tapping frequency harmonics as derived from the approach curves of **(b)** for a similar tapping amplitude as in Fig. 2.

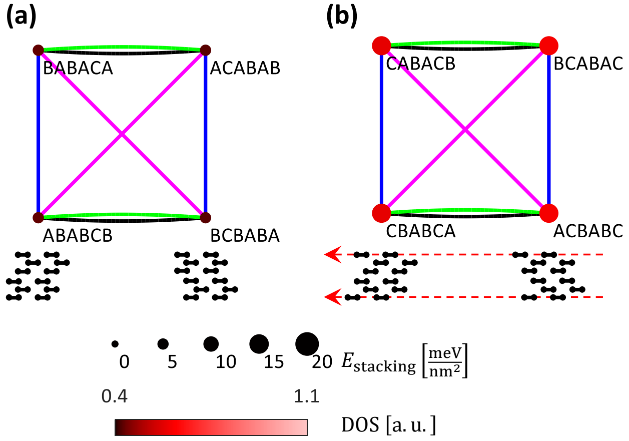

Supplementary Fig. 3. Exploration of additional TDTG moiré superlattice structures. **(a-b)** Each panel addresses a group of TDTG configurations which were not discussed in the main text. Each moiré-pair is connected by a horizontal black line. Configurations that are C_2 symmetry pairs are connected by blue (C_2^x), green (C_2^z) and magenta ($C_2^x C_2^z$) lines. Each configuration is marked by a circle whose color indicates Fermi level spectral weight, and whose size indicates the configuration's stacking energy density (see legend). Inset - bottom: Schematics of the configurations. The red arrow indicates the required global layer sliding in order to realize the particular moiré superlattice from the nominal ABABCB/BCBABA configuration.

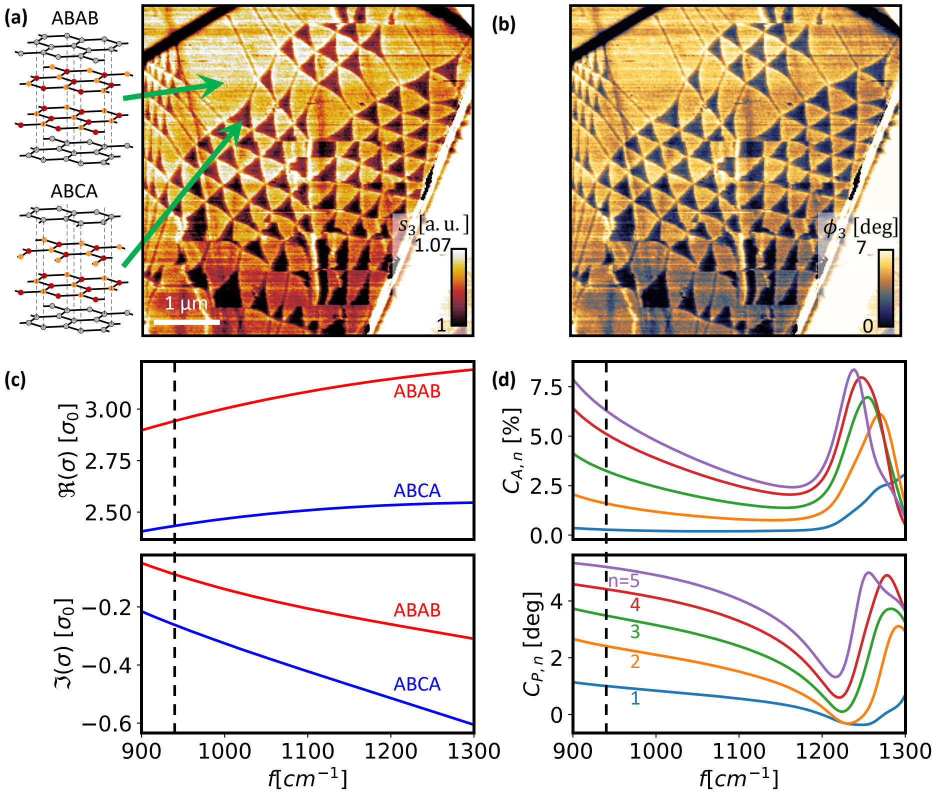

Supplementary Fig. 4. **Modelling SNOM contrast in TDBG**. (**a** and **b**) Near-field amplitude and phase scans of a marginally twisted TDBG sample, acquired at 940 cm^{-1} in the third tapping harmonic. Triangular domains correspond to ABAB (light) and ABCA (dark) stacking configurations, as illustrated on the left. **(c)** Real (top) and imaginary (bottom) parts of the frequency dependent optical conductivities of ABAB (red) and ABCA (blue) graphene derived from tight binding calculations of the band structures, plotted in units of $\sigma_0 = \frac{\pi e^2}{2h}$. **(d)** LRM calculations of the frequency dependent near-field contrast between ABAB and ABCA domains for the lowest five tapping harmonics, n. Amplitude and phase contrasts are shown in top and bottom plots, respectively. Vertical dashed line indicates the frequency at which (a) and (b) were acquired. LRM calculations assume a tip with 30 nm radius of curvature and 40 nm tapping amplitude above the surface of ABAB or ABCA graphene on hBN (40 nm thick) on SiO_2 (285 nm) on a Si substrate.
